# *QuickStats:* Age-Adjusted Percentage* of Adults Aged ≥18 Years with Hypertension,^†^ by Sex and Race and Ethnicity — United States, August 2021–August 2023

**DOI:** 10.15585/mmwr.mm7348a5

**Published:** 2024-12-05

**Figure:**
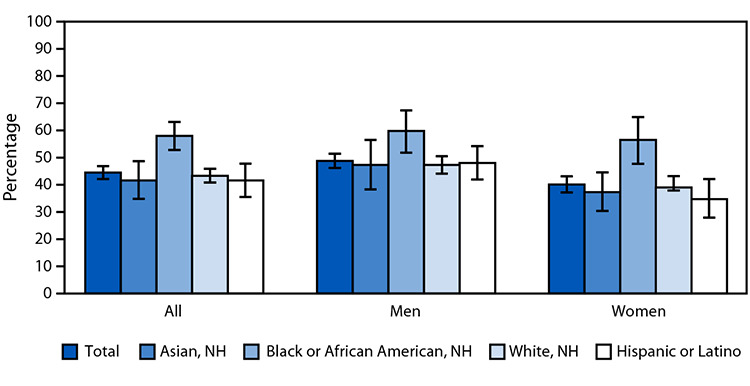
During August 2021–August 2023, the age-adjusted percentage of adults with hypertension was 44.5% and was highest among non-Hispanic Black or African American (Black) adults (58.0%). Hypertension was also highest for Black adults among both men and women. In addition, hypertension was higher among non-Hispanic Asian, non-Hispanic White, and Hispanic or Latino men compared with women.

**Supplementary Table:** https://stacks.cdc.gov/view/cdc/170362

**Source:** National Center for Health Statistics, National Health and Nutrition Examination Survey, August 2021–August 2023. https://www.cdc.gov/nchs/nhanes/index.htm

For more information on this topic, CDC recommends the following link: https://www.cdc.gov/high-blood-pressure/about/index.html

